# LRRK2 phosphorylation level correlates with abnormal motor behaviour in an experimental model of levodopa-induced dyskinesias

**DOI:** 10.1186/s13041-016-0234-2

**Published:** 2016-05-11

**Authors:** Jennifer Stanic, Manuela Mellone, Maria Daniela Cirnaru, Maria Perez-Carrion, Elisa Zianni, Monica Di Luca, Fabrizio Gardoni, Giovanni Piccoli

**Affiliations:** DiSFeB, Dipartimento di Scienze Farmacologiche e Biomolecolari, Università degli Studi di Milano, Milano, Italy; IN-CNR, Milano, Italy; Dulbecco Telethon Institute and Center for Integrative Biology (CIBIO), University of Trento, Trento, Italy

**Keywords:** Parkinson’s disease, 6-OHDA, L-DOPA, Rat, L-DOPA-induced dyskinesias, LRRK2, Phosphorylation

## Abstract

Levodopa (L-DOPA)-induced dyskinesias (LIDs) represent the major side effect in Parkinson’s disease (PD) therapy. Leucine-rich repeat kinase 2 (LRRK2) mutations account for up to 13 % of familial cases of PD. LRRK2 N-terminal domain encompasses several serine residues that undergo phosphorylation influencing LRRK2 function. This work aims at investigating whether LRRK2 phosphorylation/function may be involved in the molecular pathways downstream D1 dopamine receptor leading to LIDs. Here we show that LRRK2 phosphorylation level at serine 935 correlates with LIDs induction and that inhibition of LRRK2 induces a significant increase in the dyskinetic score in L-DOPA treated parkinsonian animals. Our findings support a close link between LRKK2 functional state and L-DOPA-induced abnormal motor behaviour and highlight that LRRK2 phosphorylation level may be implicated in LIDs, calling for novel therapeutic strategies.

## Background

Parkinson’s disease (PD) is a neurodegenerative disorder characterized by the progressive loss of dopaminergic neurons from the substantia nigra pars compacta, resulting in a decrease of dopamine (DA) levels in the striatum. Up-to-date, DA replacement with L-3,4-dihydroxyphenylalanine methyl ester hydrochloride (L-DOPA) is the most effective therapeutic approach for PD. Unfortunately, as the disease progresses, L-DOPA administration is responsible for severe side effects known as L-DOPA-induced dyskinesias (LIDs) [1]. The most parsimonious explanation of LIDs involves overactivation of D1 dopamine receptor (D1R) and extracellular signal regulated kinases 1 and 2 (ERK1/2) [1–3].

Leucine-rich repeat kinase 2 (LRRK2) mutations account for up to 13 % of familial PD cases compatible with dominant inheritance [4, 5] and have been identified in 1–2 % of sporadic PD patients [6]. LRRK2 is a large protein encompassing several functional domains including a kinase domain similar to mitogen activated protein kinase kinase kinases (MAPKKK) and N- and C-terminal protein interaction domains [7, 8]. LRRK2 is phosphorylated in vitro by a variety of serine/threonine kinases, including protein kinase C (PKC) zeta, serine protein kinase ataxia telangiectasia mutated, the IκB kinase family, and c*yclic adenosine monophosphate*-dependent protein kinase (PKA) [9]. Accumulating evidence suggest that Ser 910 and 935 within LRRK2 N-terminal domain undergo dynamic activity-dependent phosphorylation/dephosphorylation cycle. In particular, LRRK2 is phosphorylated on Ser-935/910 by PKA [10], and, at the same time, it regulates PKA signalling to the ERK1/2 pathway [11]. Notably, it has also been suggested that LRRK2 influences synaptogenesis and D1R and GluR1 signalling via PKA [11]. In this study we hypothesize that LRRK2 may be involved in the molecular pathway downstream D1R activation leading to LIDs.

## Results

**Fig. 1:**
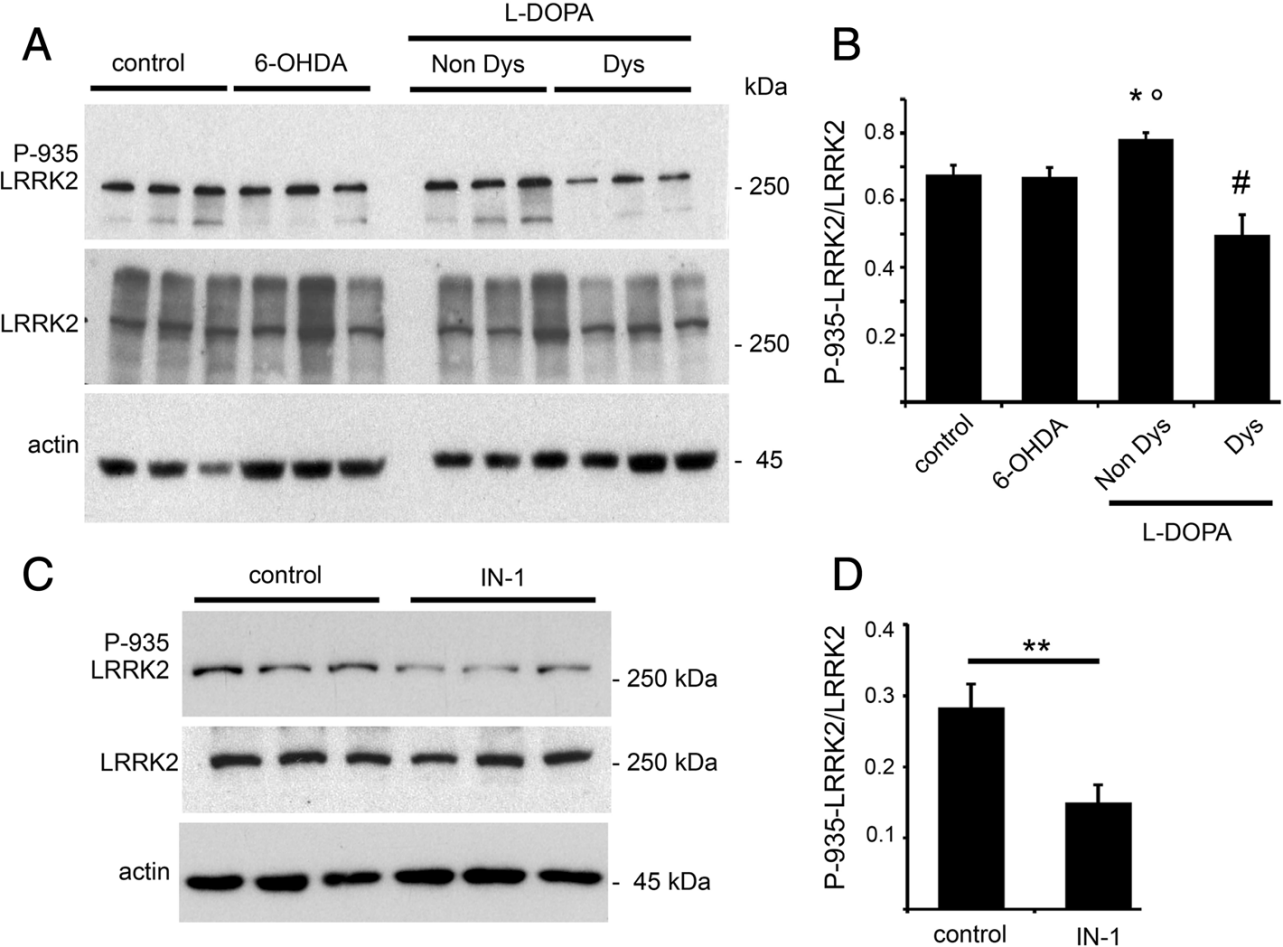
LRRK2 phosphorylation at Ser-935 in parkinsonian and dyskinetic rats. **a** The ipsilateral striatum from non lesioned (control), fully lesioned parkinsonian (6-OHDA), non dyskinetic (Non Dys) rats or animals displaying a dyskinetic behaviour (Dys) were analyzed by WB to evaluate the levels of LRRK2 phosphorylation at Ser-935 site (P-935). **b** The graph illustrates LRRK2 phosphorylation normalized upon total LRRK2 (*n* = 6; One-way Anova: *p < 0.05 Non Dys versus 6-OHDA °*p* < 0.01 Non dys versus Dysk, #*p* < 0.05, Dys versus 6-OHDA). Data are expressed as mean ± SEM. **c** Rats were injected in the left striatum with LRRK2 IN-1 inhibitor or vehicle (control) and the total and phosphorylated LRRK2 levels were evaluated by WB. **d** IN-1 reduced LRRK2 phosphorylation in vivo (*n* = 6; **p < 0.01, unpaired two-tailed Student’s *t*-test). Graph reports LRRK2 phosphorylation level normalized on the total amount of LRRK2 protein. Data are expressed as mean ± SEM

Given the tight functional correlation between LRRK2 and crucial LIDs molecular players, we measured LRRK2 expression and phosphorylation levels in the striatum from control, 6-OHDA and L-DOPA-treated non dyskinetic and dyskinetic rats. Interestingly, while LRRK2 total expression was unaltered in the different experimental groups, LRRK2 phosphorylation at Ser-935 (P-935; Fig. 1a-b) was significantly modified following treatment with L-DOPA. In particular, dyskinetic animals were characterized by a significant decrease in P-935 tone compared to non dyskinetic rats. Conversely, non dyskinetic rats were characterized by a significantly higher P-935 level compared to 6-OHDA animals (Fig. 1a-b).

Based on the above-described molecular results, we questioned whether LRRK2 phosphorylation level could directly correlate with LIDs onset. To prove this hypothesis, we first evaluated the efficacy of LRRK2 inhibitors in vivo. To this, we injected in the left striatum of naive rats 5 nmol of IN-1 inhibitor [12] or its vehicle (0.5 % DMSO). The treatment with either DMSO or IN-1 did not influence the motor behaviour of naive animals (data not shown). After 2 h, we sacrificed the animals and removed the ipsi and controlateral striata. Next, we processed striatal specimen for biochemical investigation. Indeed, IN-1 reduced LRRK2 phosphorylation at Ser-935 in vivo (Fig. 1c-d). LRRK2 inhibitor III was already described as capable to pass the blood brain barrier (BBB) and inhibit LRRK2 in the central nervous system [13].

**Fig. 2:**
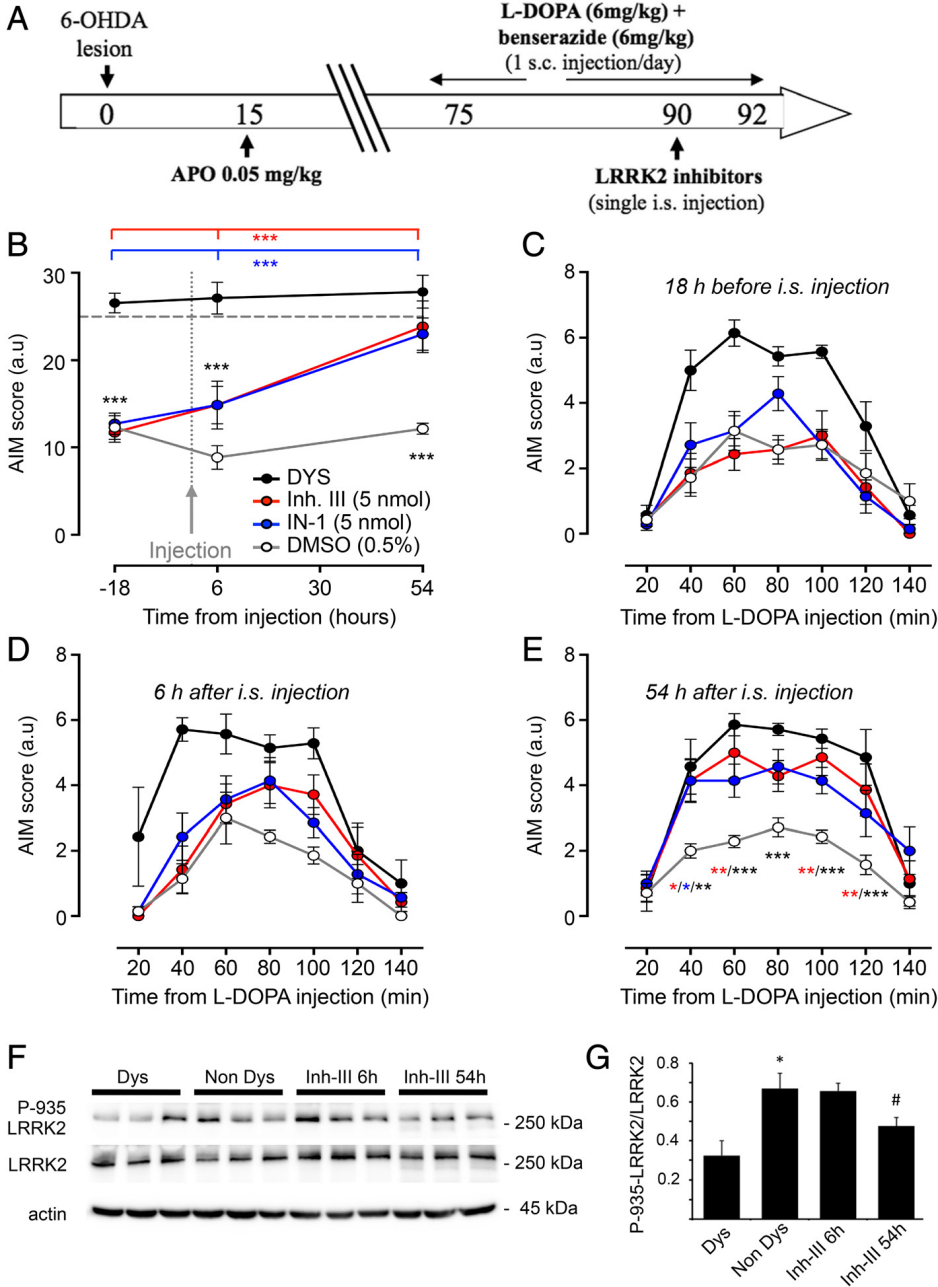
Effects of LRRK2 inhibitors on dyskinesia in 6-OHDA parkinsonian rats. **a** The panel is a schematic representation of the experimental design. Rats were unilaterally injected with 6-OHDA in the left MFB. After 15 days, the extent of the lesion was evaluated upon apomorphine answer. Two months after the 6-OHDA injection, fully lesioned rats (>200 controlateral turns) were chronically treated with L-DOPA. LRRK2 inhibitors were administrated to low dyskinetic animals with a single intrastriatal (i.s.) injection after 2 weeks of L-DOPA treatment. LIDs onset and severity were evaluated by AIMs scoring. **b** IN-1 (5 nmol), LRRK2 inhibitor III (5 nmol) or vehicle (5 μl 0.5 % DMSO) were stereotaxically injected in the ipsilateral striatum of low dyskinetic rats. I.s. injections were performed 6 h before the daily L-DOPA administration and the evaluation of AIMs were carried out from 20 to 140 min after L-DOPA. Both inhibitors were able to induce a significant increase in the AIMs score compared to vehicle injected rats and to pre-surgery values (*n* = 7; −18 h: ***p < 0.001, DYS vs DMSO/IN-1/Inh. III. 6 h: ***p < 0.001, DYS vs DMSO/IN-1/Inh. III. 54 h: ***p < 0.001, DMSO vs IN-1/Inh. III/DYS. IN-1: ***p < 0.001, 54 h vs −18 h/6 h. Inhibitor III: ***p < 0.001, 54 h vs −18 h/6 h vs 54 h; two-way ANOVA followed by Bonferroni *post-hoc* test). **c-e** Both inhibitors significantly increased dyskinesia induction, as indicated by AIMs scoring during the single last observation session (54 h; E) (*n* = 7; 40 min, DMSO vs IN-1/Inh. III: *p < 0.05, DMSO vs DYS: **p < 0.01; 60 min, DMSO vs Inh. III: **p < 0.01, DMSO vs DYS: ***p < 0.001; 80 min, DMSO vs DYS: ***p < 0.001; 100 min, DMSO vs Inh. III: **p < 0.01, DMSO vs DYS: ***p < 0.001; 120 min, DMSO vs Inh. III: **p < 0.01, DMSO vs DYS: ***p < 0.001; two-way ANOVA plus Bonferroni *post-hoc* test). **f** The ipsilateral striatum from rats displaying a dyskinetic behaviour (Dys), non dyskinetic rats (Non Dys), Non Dys animals sacrificed 6 h after Inh-III injection (Inh-III 6 h) or Non Dys animals sacrificed 54 h after Inh-III injection (Inh-III 54 h) were analyzed by WB to evaluate the levels of LRRK2 phosphorylation at Ser-935 site (P-935). **g** The graph illustrates LRRK2 phosphorylation normalized upon total LRRK2 (*n* = 3; One-way Anova: *p < 0.01 Non Dys versus Dys #*p* < 0.05 Non Dys versus Inh-III 54 h). Data are expressed as mean ± SEM

Considering that LRRK2 phosphorylation level inversely correlates with LIDs (Fig. 1a-b), we verified if administration of IN-1 and LRRK2 inhibitor III to rats without a dyskinetic profile could affect their motor behaviour. Therefore, IN-1 (5 nmol), LRRK2 inhibitor III (5 nmol) or vehicle (5 μl 0.5 % DMSO) were injected in the ipsilateral striatum of 6-OHDA-lesioned rats displaying a low dyskinetic behaviour following L-DOPA chronic treatment (15 days; Fig. 2a). Intrastriatal injections were performed 6 h before the daily injection of L-DOPA. As shown in Fig. 2b, both inhibitors were able to induce a significant and similar increase in the total AIMs score compared to vehicle injected rats and pre-surgery scores. Moreover, the time course of AIMs development (Fig. 2c-e) indicated a more pronounced effect of LRRK2 inhibitor III compared to IN-1 in AIMs induction, as measured during the single last observation session (Fig. 2e). Accordingly, western-blotting analysis revealed a significant decrease of Ser-935 phosphorylation in animals sacrificed 54 h after inhibitor III injection (Fig. 2f-g).

## Discussion

In the last decades, the understanding of the molecular mechanisms underlying LIDs onset has greatly advanced [1]. However, there is still a relevant discrepancy between such knowledge and the availability of therapeutic strategies able to prevent or ameliorate LIDs. Here we show that LRRK2 phosphorylation level is increased in non dyskinetic rats and that pharmacological inhibition of LRRK2 worsens the dyskinetic motor behaviour in parkinsonian animals chronically treated with L-DOPA.

Besides its potential role at the presynaptic site [14–17], LRRK2 has been implicated in the morphological and functional maturation of dendritic spines. In particular, it has been suggested that LRRK2 influences synaptogenesis and D1R and GluR1 signalling via PKA [11]. Furthermore, we have recently demonstrated that LRRK2 interacts with and phosphorylates N-ethylmaleimide sensitive factor (NSF) [17]. NSF is relevant not only for vesicle recycling at the presynaptic bouton, but it is also crucial to allow proper expression of α-amino-3-hydroxy-5-methyl-4-isoxazolepropionic acid (AMPA) and D1R receptors at the postsynaptic membrane [18]. In particular, PKC γ-induced trafficking of AMPA receptors depends on NSF and PICK1 [19]. Overall, it could be argued that LRRK2 might influence the composition and the functionality of synaptic receptor repertoire expressed on striatal neurons.

LRRK2 activity is tightly regulated by a still unresolved balance between auto/heterophosphorylation. Serine phosphorylation is required for binding to 14-3-3. Dephosphorylation results in LRRK2:14-3-3 complex dissociation [20, 21] with subsequent LRRK2 relocalization into defined cellular compartments including cytoskeletal elements and centrosomes [20, 22]. Several kinases, including PKA and PKCz, phosphorylate LRRK2 at Ser-935 in vitro, thus potentially positioning LRRK2 within the signalling cascade triggered by D1R/D2R. Given the impact of LRRK2 not only on PKA and NSF but also on ERK1/2 pathway, we believe that D1R/D2R activity influences receptor expression and output acting via LRRK2. Finally, notwithstanding one previous study on levodopa-treated Israeli PD patients reported no over effect of G2019S mutation on LID prevalence or latency [23], it will be of great relevance the investigation of LID development in LRRK2 transgenic models, including loss-of-function and knock-in.

## Conclusions

Our data suggest that the molecular alteration underlying LIDs could impinge on LRRK2 and that the establishment of treatments restoring/preserving proper LRRK2 phosphorylation level would be of great therapeutic value for LIDs prevention.

## Material and methods

### Animals

Adult male Sprague Dawley rats (Charles River Laboratories, Calco, Italy) were maintained on a 12-h light/dark cycle at 22 °C with food and water ad libitum. Procedures were carried out in accordance with the current European Law (Directive 2010/63/EU).

### Antibodies

The primary antibodies used in this study are rabbit anti-LRRK2 1:500 MJFF C41-2, rabbit anti-LRRK2 P-Ser-935 UDD2 10(12) (Abcam, Cambridge, UK), mouse anti-actin 1:1000, mouse anti-FLAG 1:1000, mouse anti myc 1:1000, mouse anti-synaptophysin 1:1000 (Sigma-Aldrich St. Louis, MO, USA).

### 6-hydroxydopamine (6-OHDA) rat model

Rats (*n* = 100; 125–175 g) underwent a single stereotaxic injection of 6-OHDA (Sigma-Aldrich, St. Louis, MO, USA; 12 μg/4 μl, rate of injection 0.38 μl/min) in the left medial forebrain bundle (MFB; AP: +4.4, L: −1.2; DV: −7.5) as previously described [24, 25]. Fifteen days after the lesion, the rats were challenged with 0.05 mg/kg apomorphine (Sigma-Aldrich; s.c. injection) to assess the entity of the lesion. Rats with a complete DA denervation (fully lesioned rats) were enrolled in the study.

### L-DOPA-induced dyskinesias (LIDs) and treatment with LRRK2 inhibitors

Two months after the 6-OHDA injection, fully lesioned rats were treated with 6 mg/kg L-DOPA and 6 mg/kg benserazide (1 s.c. injection/day) for 14 days. L-DOPA-induced abnormal involuntary movements (AIMs) were evaluated on days 4, 7, 10 and 14 of L-DOPA administration using a highly validated rat AIMs scale [25, 26]. In brief, rats were observed individually for 1 min every 20 min from 20 to 140 min after the daily L-DOPA injection. At each observation time point the AIMs were classified into 3 subtypes: i. axial (dystonic or choreiform torsion of the upper part of the body toward the side contralateral to the lesion), ii. limb (jerky and/or dystonic movements of the forelimb contralateral to the lesion) and iii. orolingual (empty jaw movements and tongue protrusion). Each subtype was scored on a severity scale from 0 to 4 (0 = absent, 1 = present during less than half of the observation time (<30 s), 2 = present for more than half of the observation time (>30 s), 3 = present for 1 min but suppressible by external stimuli, 4 = present all the time but not suppressible by external stimuli). The total AIMs score for each test session was obtained by summing the scores of all observation time points. Animals were then divided in 2 groups according to their AIMs score: rats with low (AIMs <15) and high (AIMs > 25) dyskinetic behaviour.

At day 15 of L-DOPA administration, fully lesioned rats showing a low dyskinetic behaviour underwent a single stereotaxic injection of 2 LRRK2 inhibitors IN-1 (5 nmol; *n* = 7) and Inhibitor III (5 nmol; *n* = 7), or 0.5 % DMSO (*n* = 7) in the ispilateral striatum (AP = +0.2, L = +3.5, DV = −5.7; rate of injection 0.5 μl/min). Untreated fully lesioned animals displaying stable and severe dyskinesia (AIMs >25; *n* = 7) were included in the study as positive control. L-DOPA treatment was continued for 4 days after injection of LRRK2 inhibitors. To evaluate the effects of LRRK2 inhibition on dyskinesia, AIMs assessment on IN-1/InhibIII/DMSO-treated rats was carried out before the surgery (−18 h), on the day of the surgery and after 2 days (6 and 54 h after the injection, respectively).

### Western blot (WB)

Striata from 6-OHDA and L-DOPA-treated non dyskinetic and dyskinetic rats were dissected and solubilized in 150 mM NaCl, 50 mM Tris pH 7.4, 1 % v/v NP-40, 0.1 % v/v SDS with protease and phosphatase inhibitors. The contralateral striatum from 6-OHDA rats was used as control. Proteins were separated by SDS-PAGE, transferred onto a nitrocellulose membrane and incubated with the appropriate primary and secondary antibodies. Labeling was visualised by ECL chemiluminescence detection system and quantification was performed by densitometric analysis of the fluorograms (Quantity One software, Bio-Rad, Hercules, CA, USA).

### Statistical analysis

All data are expressed as mean ± SEM. Significance of the differences was analysed by unpaired two-tailed Student’s t test or ANOVA followed by Dunn’s and Bonferroni *post hoc* test as indicated in the text.

## Ethical approval

Not applicable.

## Consent for publication

Not applicable.

## Availability of data and materials

The datasets supporting the conclusions of this article are available in the Open Science Framework repository at https://osf.io/u4nzd/.

## Abbreviations

6-OHDA: 6-hydroxydopamine
AIMs: abnormal involuntary movements
AMPA receptor: α-amino-3-hydroxy-5-methyl-4-isoxazolepropionic acid receptor
D1R: D1 dopamine receptor
D2R: D2 dopamine receptor
DA: dopamine
DMSO: dimethyl sulfoxide
ECL: enhanced chemiluminescence
ERK1/2: extracellular signal regulated kinases 1 and 2
i.s.: intrastriatal
L-DOPA: L-3,4-dihydroxyphenylalanine methyl ester hydrochloride
LIDs: L-DOPA-induced dyskinesia
LRRK2: leucine-rich repeat kinase 2
MAPKKK: mitogen activated protein kinase kinase kinases
MBF: medial forebrain bundle
NP-40: nonidet P-40
PKA: cyclic adenosine monophosphate-dependent protein kinase
s.c.: subcutaneous
SDS-PAGE: Sodium Dodecyl Sulphate-PolyAcrylamide Gel Electrophoresis
SEM: standard error of the mean

## References

[1] Bastide MF, Meissner WG, Picconi B, Fasano S, Fernagut P-O, Feyder M (2015). Pathophysiology of L-dopa-induced motor and non-motor complications in Parkinson’s disease. Prog Neurobiol.

[2] Feyder M, Bonito-Oliva A, Fisone G (2011). L-DOPA-induced dyskinesia and abnormal signaling in striatal medium spiny neurons: focus on dopamine D1 receptor-mediated transmission. Front Behav Neurosci.

[3] Santini E, Valjent E, Usiello A, Carta M, Borgkvist A, Girault J-A (2007). Critical involvement of cAMP/DARPP-32 and extracellular signal-regulated protein kinase signaling in L-DOPA-induced dyskinesia. J Neurosci.

[4] Paisán-Ruíz C, Paisán-Ruíz C, Jain S, Evans EW, Gilks WP, Simón J (2004). Cloning of the gene containing mutations that cause PARK8-linked Parkinson’s disease. Neuron.

[5] Zimprich A, Biskup S, Leitner P, Lichtner P, Farrer M, Lincoln S (2004). Mutations in LRRK2 cause autosomal-dominant parkinsonism with pleomorphic pathology. Neuron.

[6] Berg D (2005). Type and frequency of mutations in the LRRK2 gene in familiar and sporadic Parkinson’s disease. Brain.

[7] Bosgraaf L, Van Haastert PJM (2003). Roc, a Ras/GTPase domain in complex proteins. Biochim Biophys Acta.

[8] Guo L, Wang W, Chen SG (2006). Leucine-rich repeat kinase 2: relevance to Parkinson’s disease. Int J Biochem Cell Biol.

[9] Wallings R, Manzoni C, Bandopadhyay R (2015). Cellular processes associated with LRRK2 function and dysfunction. FEBS J.

[10] Muda K, Bertinetti D, Gesellchen F, Hermann JS, von Zweydorf F, Geerlof A (2014). Parkinson-related LRRK2 mutation R1441C/G/H impairs PKA phosphorylation of LRRK2 and disrupts its interaction with 14-3-3. Proc Natl Acad Sci U S A.

[11] Parisiadou L, Yu J, Sgobio C, Xie C, Liu G, Sun L (2014). LRRK2 regulates synaptogenesis and dopamine receptor activation through modulation of PKA activity. Nat Neurosci.

[12] Deng X, Dzamko N, Prescott A, Davies P, Liu Q, Yang Q (2011). Characterization of a selective inhibitor of the Parkinson’s disease kinase LRRK2. Nat Chem Biol.

[13] Choi HG, Zhang J, Deng X, Hatcher JM, Patricelli MP, Zhao Z (2012). Brain penetrant LRRK2 inhibitor. ACS Med Chem Lett.

[14] Piccoli G, Condliffe SB, Bauer M, Giesert F, Boldt K, De Astis S (2011). LRRK2 controls synaptic vesicle storage and mobilization within the recycling pool. J Neurosci.

[15] Piccoli G, Onofri F, Cirnaru M-D, Kaiser CJO, Jagtap P, Kastenmüller A (2014). Leucine-rich repeat kinase 2 binds to neuronal vesicles through protein interactions mediated by its C-terminal WD40 domain. Mol Cell Biol.

[16] Cirnaru MD, Marte A, Belluzzi E, Russo I, Gabrielli M, Longo F (2014). LRRK2 kinase activity regulates synaptic vesicle trafficking and neurotransmitter release through modulation of LRRK2 macro-molecular complex. Front Mol Neurosci.

[17] Belluzzi E, Gonnelli A, Cirnaru M-D, Marte A, Plotegher N, Russo I (2016). LRRK2 phosphorylates pre-synaptic N-ethylmaleimide sensitive fusion (NSF) protein enhancing its ATPase activity and SNARE complex disassembling rate. Mol Neurodegener.

[18] Chen S, Liu F (2010). Interaction of dopamine D1 receptor with N-ethylmaleimide-sensitive factor is important for the membrane localization of the receptor. J Neurosci Res.

[19] Patten SA, Ali DW (2009). PKCgamma-induced trafficking of AMPA receptors in embryonic zebrafish depends on NSF and PICK1. Proc Natl Acad Sci U S A.

[20] Nichols RJ, Dzamko N, Morrice NA, Campbell DG, Deak M, Ordureau A (2010). 14-3-3 binding to LRRK2 is disrupted by multiple Parkinson’s disease-associated mutations and regulates cytoplasmic localization. Biochem J.

[21] Dzamko N, Deak M, Hentati F, Reith AD, Prescott AR, Alessi DR (2010). Inhibition of LRRK2 kinase activity leads to dephosphorylation of Ser 910/Ser 935, disruption of 14-3-3 binding and altered cytoplasmic localization. Biochem J.

[22] Mamais A, Chia R, Beilina A, Hauser DN, Hall C, Lewis PA (2014). Arsenite stress down-regulates phosphorylation and 14-3-3 binding of leucine-rich repeat kinase 2 (LRRK2), promoting self-association and cellular redistribution. J Biol Chem.

[23] Yahalom G, Kaplan N, Vituri A, Cohen OS, Inzelberg R, Kozlova E (2012). Dyskinesias in patients with Parkinson’s disease: effect of the leucine-rich repeat kinase 2 (LRRK2) G2019S mutation. Parkinsonism Relat Disord.

[24] Picconi B, Paillé V, Ghiglieri V, Bagetta V, Barone I, Lindgren HS (2008). l-DOPA dosage is critically involved in dyskinesia via loss of synaptic depotentiation. Neurobiol Dis.

[25] Gardoni F, Picconi B, Ghiglieri V, Polli F, Bagetta V, Bernardi G (2006). A critical interaction between NR2B and MAGUK in L-DOPA induced dyskinesia. J Neurosci.

[26] Lundblad M, Andersson M, Winkler C, Kirik D, Wierup N, Cenci MA (2002). Pharmacological validation of behavioural measures of akinesia and dyskinesia in a rat model of Parkinson’s disease. Eur J Neurosci.

